# Preclinical Evaluation and Monitoring of the Therapeutic Response of a Dual Targeted Hyaluronic Acid Nanodrug

**DOI:** 10.1155/2017/4972701

**Published:** 2017-07-11

**Authors:** Minglong Chen, Wenqi Zhang, Kai Yuan, Mingxiang Bo, Bin Chen, Lu Li, Qingjie Ma, Lei Zhu, Shi Gao

**Affiliations:** ^1^Department of Nuclear Medicine, China-Japan Union Hospital, Jilin University, Changchun 130033, China; ^2^Department of Breast Surgery, Shandong Provincial Qianfoshan Hospital, Shandong University, Jinan 250012, China; ^3^Departments of Surgery, Emory University School of Medicine, Atlanta, GA 30322, USA; ^4^Pharmaceutical Department, Binzhou Medical University Hospital, Binzhou 256603, China

## Abstract

Chemotherapy is a powerful cancer treatment but suffers from poor biocompatibility and a lack of tumor targeting. Here, we developed a CD44-targeted polymeric nanocomplex by encapsulating 10-hydroxycamptothecin (HCPT) into hyaluronic acid nanoparticles (HANP) for targeted cancer therapy. In vitro, the HANP/HCPT showed improved cytotoxicity to five cancer cell lines including HT29, A549, MDA-MB-231, HepG2, and MDA-MB-435 versus free HCPT. After systemic administration into MDA-MB-231 breast cancer xenograft, tumor growth was significantly inhibited 5.25 ± 0.21 times in the HANP/HCPT treated group relative to the nontreated group. In addition, the treatment response was also accessed and confirmed by 18F-fluoro-2-deoxy-D-glucose ([18F] FDG) positron emission tomography (PET). The MDA-MB-231 tumors responded to HANP/HCPT 7 days after the first treatment, which benefits treatment strategy adjustment and personalization. No apparent systemic toxic effects were seen in mice treated with HANP/HCPT. In summary, the HANPs have great promise as a targeted drug carrier for cancer chemotherapy. Our HANP platform can also deliver other hydrophobic chemotherapy agents.

## 1. Introduction

Cancer is a major public health problem worldwide and is the second leading cause of death in the United States. In 2017, 1,688,780 new cancer cases and 600,920 cancer deaths are projected to occur in the United States [1]. Chemotherapy modality is one of the most conventional therapeutic regimes to ablate tumor growth [2, 3]. Of the chemotherapy agents, 10-hydroxycamptothecin (HCPT), is a broad spectrum antitumor agent against many solid tumors in animal models and patients [4–6]. Although camptothecin (CPT) inhibits cancer cells by the same mechanism as HCPT, HCPT is more active and less toxic [5]. However, poor water solubility and tumor targeting still limit the clinical applications of HCPT.

Nanotechnology can help optimize pharmacokinetic profiles and in vivo distribution of the conventional chemotherapy drugs. This can reduce the systemic toxicity and improve the tumor therapeutic effects [7]. Polymer nanoparticles are promising drug carriers with excellent biocompatibility and biodegradability [8, 9]. These polymer nanoparticles are prepared via a self-assembly strategy using amphiphilic block copolymers in aqueous media. They are generally comprised of a hydrophilic outer shell and a hydrophobic inner core that can incorporate lipophilic drugs into their cores and later release the drug in a controlled manner. This makes them a promising carrier for drugs with poor water solubility.

Specifically, hyaluronic acid- (HA-) based polymeric nanoparticles have attracted great attention in drug delivery as well as photodynamic therapy, photothermal therapy, and chemotherapy. As we reported, the HA was chemically modified with a hydrophobic moiety such as 5*β*-cholanic acid (5*β*-CA). It can self-assemble into nanoparticles under physiological conditions with a hydrophilic surface and hydrophobic cavities to offer ligand modification and encapsulation of poorly biocompatible agents [10–12]. The HA can specifically bind to specific cell surface receptors like cluster determinant 44 (CD44) or lymphatic vessel endothelial hyaluronan receptor- (LYVE-) 1, which are overexpressed in various cancer cells [13, 14]. Thus, the HANP demonstrated improved tumor targeting via active targeting and passive enhanced permeation and retention (EPR) effects. We and others have shown that the HANP system can be used as effective vehicles for successful treatment of tumors [8, 10]; however, the accurate evaluation of tumor treatment response has not yet been studied, and the tumor response to treatment with HANP-based drugs remains unclear in vivo.

Monitoring treatment can identify responding and nonresponding tumors and help doctors adjust the therapeutic regimen and improve the efficacy of standard chemotherapy [15, 16]. Traditional methods to assess therapeutic effects use changes in tumor size and clinical symptoms but are slow and inaccurate. An effective treatment might cause physiological and biochemical changes in the tumor prior to changes in tumor size. It is now possible to predict the therapeutic response via imaging. Positron emission tomography (PET) can measure molecular pathways in vivo and can characterize multiple aspects of oncologic pathology including metabolism, angiogenesis, cellular proliferation, and blood flow [17–21]. Molecular imaging by PET/CT with specific functional probes can provide highly sensitive imaging with high resolution (1-2 mm spatial resolution) [22], and it can evaluate biological and metabolic activity status of tumor cells, which can facilitate comprehensive evaluation of tumors and improve early detection, staging, and monitoring of therapeutic responses [19]. The 18F-fluoro-2-deoxy-D-glucose ([18F] FDG) is a glucose analog taken up into tumor cells by glucose transporters, which are overexpressed in tumors and imaged with PET [23, 24]. It has been successfully used to monitor the response of chemotherapy [25], but PET is rarely used with nanomaterial-based drugs.

In this study, we encapsulated the conventional chemotherapy drug, 10-hydroxycamptothecin (HCPT), into HANP and formed a tumor-specific complex for chemotherapy: HANP/HCPT. We measured 37.08 ± 1.54% HCPT loaded into HANP with 92.7% efficiency when the HCPT and HANP ratios were 2 : 3. The targetability and therapeutic efficiency of HANP/HCPT were then evaluated in vitro, and they demonstrated improved cytotoxicity to cancer cells compared to free HCPT.

After intravenous administration, the HANP/HCPT significantly inhibited tumor growth with minimal organ toxicity. Furthermore, the antitumor effects were evaluated and confirmed with [18F] FDG PET. The tumor ablation effects were detected as early as 7 days after the first HANP/HCPT injection. Overall, the HANP/HCPT has excellent biocompatibility and reduced the systematic toxicity of the drug, which is promising for clinical applications. The use of [18F] FDG PET confirmed the activity of HANP/HCPT; PET is useful in the clinic and can help physicians tune their dose guidelines. The HANP system can also be extended for other hydrophobic small molecules and targeted therapy.

## 2. Materials and Methods

### 2.1. Materials

Sodium hyaluronate (MW = 2.344 × 105) was used after being dialyzed against distilled water, followed by freeze-drying. 5*β*-Cholanic acid (CA), tetrabutylammonium hydroxide (TBA), and propidium iodide (PI) were obtained from Sigma-Aldrich Co. (St. Louis, MO, USA). 10-Hydroxycamptothecin (HCPT) was obtained from Bioengineering Co., Ltd. (Shanghai, China). Water used for synthesis and characterization was purified by distillation, deionization, and reverse osmosis. HT29 (colorectal cancer cells), A549 (lung adenocarcinoma), MDA-MB-231 (breast cancer cells), HepG2 (hepatocellular carcinoma), MDA-MB-435 (breast cancer cells), and NIH-3T3 (mouse embryonic fibroblast cells) were purchased from ATCC (Manassas, VA). MDEM was obtained from Thermo scientific (MA, USA). Hyaluronidase, MTT assay kit, and 4,6-diamidino-2-phenylindole (DAPI) were purchased form Bioengineering Co., Ltd. (Shanghai, China). All other chemicals were of analytical grade and used without further purification.

### 2.2. Preparation and Characterization of Drug-Loaded HANPs

HANPs was prepared by high pressure homogenizer (PhD Technology International LLC, USA). In brief, hyaluronic acid (HA) was converted to the tetrabutylammonium salt of HA (HA-TBA) using a previously reported method [26]. Next, HA-5*β*-cholanic acid (HACA) conjugate was synthesized by linking the carboxyl group of HA-TBA with the amino group on CA in the presence of EDC and NHS. At last, HANP/HCPT were prepared under high pressure homogenizer. In brief, HANPs (80 mg) were dispersed in 16 mL of distilled water and HCPT (20 mg) were dissolved in 2 mL dimethyl sulfoxide (DMSO) and dimethylformamide (DMF). The HCPT solution was slowly added to the HANP in high pressure homogenizer. The resulting mixture was dialyzed for 4 hours against an excess amount of distilled water to remove unloaded drugs and organic solvent, followed by lyophilization. The particle sizes of HANP and HANP/HCPT were determined using dynamic light scattering (DLS).

### 2.3. HCPT Loading Efficiency

The encapsulation efficiency of HCPT was determined by HPLC system after dissolving 1 mg/mL HANP/HCPT in distilled water (DW) and diluting with 100x the volume of the mobile phase. The drug was assayed using a Waters high-performance liquid chromatography (HPLC) system combined with a separation module, a fluorescence detector, and a reverse-phase C-18 column (5 *μ*m, 120 Å, 250 mm × 4.6 mm) using 5 to 65% acetonitrile containing 0.1% TFA versus distilled water containing 0.1% TFA over 30 min at a flow rate of 1 mL/min. Wavelength for the detection of HCPT was 254 nm. The loading efficiency of SWCNTs was calculated using the following equation: loading efficiency = *W*_loaded drug_/*W*_loaded drug_ + *W*_HANP_ × 100%

### 2.4. Storage Stability Study

The 100 *μ*L suspensions of free HCPT and HANPs/HCPT (10 mg/mL) were dispersed with 900 *μ*L DW, PBS, and DMEM in 1.5 mL EP tube, respectively. Then they were stored at room temperature. Digital photographs of the samples were taken at 0–3 days.

### 2.5. In Vitro Enzyme-Triggered Drug Release of HANP/HCPT

Drug release profiles of HANP/HCPT were determined using a dialysis method in the presence of different concentrations of hyaluronidase (Hyal). In brief, lyophilized HANP/HCPT (10 mg) were dispersed in 1 mL of phosphate-buffered saline (PBS, pH = 4.3, 37°C) with or without Hyal. The dispersed HANP/HCPT were transferred to dialysis tubes (molecular weight cutoff = 100,00 kDa) and immersed in 20 mL of PBS and gently shaken at 37°C in a water bath at 100 rpm. A 0.2 mL aliquot was collected at predetermined time points and an equal volume of fresh medium was replenished. The amount of HCPT released was determined by HPLC at 254 nm.

### 2.6. Cellular Uptake Assay

Cells were grown in DMEM supplemented with 10% heat-inactivated fetal bovine serum (FBS, Macgene Biotech) and antibiotics (penicillin 100 U/mL and streptomycin 100 mg/mL). All the cells were cultured in incubators maintained at 37°C with 5% CO_2_ in a humidified atmosphere. MDA-MB-435 cells were seeded on 6-well plates and incubated in complete medium for 24 h at 37°C. Then, the medium was replaced with fresh culture medium containing FITC conjugated HANP/HCPT (FITC-HANP/HCPT) and incubated for 4 h at 37°C. To verify the specificity of HA binding with CD44, free HA (1 mg/mL) was added to cells 30 min before HANP/HCPT. After washing with PBS (pH = 7.4) for three times, cells were fixed in cold ethanol at −20°C for 15 min. After being fixed, cells were labeled with DAPI in darkness for 10 min and then imaged by a laser scanning confocal fluorescence microscope (Leica, German) with specific filter for FITC.

### 2.7. In Vitro Cytotoxicity of HANP/HCPT

The cytotoxic effects of HCPT and HANP/HCPT were evaluated using the MTT assay. Cells were seeded into 96-well flat-bottomed plates at a density of 1.0 × 10^5^ cells/well and cultured at 37°C in a humidified atmosphere with 5% CO_2_ for 12 h. Cells were incubated in the culture medium with a series of concentrations of free HCPT (DMSO < 1%) and HANP/HCPT for 24, 48, and 72 h at 37°C. The blank culture medium was used as a blank control. The survival rate was calculated on the same day with the following formula: Survival% = (A490 nm for the treated cells/A490 nm for the control cells) × 100%, where A490 nm is the absorbance value. The dose-effect curves were plotted. All the experiments were performed in triplicated wells.

### 2.8. In Vivo Antitumor Efficacy of HANP/HCPT

To evaluate the antitumor efficacy of HANPs/HCPT, tumor-bearing mice were prepared as follows. A suspension of 5.0 × 10^6^ MDA-MB-435 cells in physiological saline (100 *μ*L) was subcutaneously injected into the dorsa of athymic nude mice (7 weeks old, 20–25 g). Mice were divided into three groups: (i) normal saline (the control group), (ii) free HCPT at 10 mg/kg, and (iii) HANP/HCPT at 10 mg HCPT/kg. When tumor volume reached 80 mm^3^, drugs were injected every three days. Tumor size was monitored and tumor volumes were calculated as *a* × *b*^2^/2, where *a* was the largest and *b* was the smallest diameter. The mouse body weight and tumor volume were measured every 3 days for up to 20 days before euthanasia. To access the therapeutic response, [18F] FDG PET was initiated on day 0 before saline, HCPT,and HANP/HCPT administration and continued to be carried out on days 3, 7, and 14, respectively. The survival rates of the mice were recorded for 30 days. Relative tumor growth rate was defined and calculated as *V*_*n*_/*V*_0_, where *V*_*n*_ is the tumor volume at day *n* and *V*_0_ is the tumor volume at day 0.

### 2.9. In Vivo PET Imaging

PET scans and image analysis were performed using an Inveon microPET scanner (Siemens Medical Solutions). At predetermined time points before (Day 0) and after PDT treatment (day 3, day 7, and day 14), each tumor-bearing mouse was injected via the tail vein with 3.7 MBq (100 mCi) of [18F] FDG in a volume of 100 *μ*L saline under isoflurane anesthesia. Five-minute static scans were acquired at 1 h after injection. Mice were kept fasting for 4 h before tracer injection and maintained under isoflurane anesthesia during the injection, accumulation, and scanning periods in the process of [18F] FDG scan. A heat pad was applied to maintain the mouse body temperature during the scanning.

### 2.10. Histological Studies

For histological analysis, animals were sacrificed after the treatments and the tumor was fixed in a 4% formaldehyde solution at room temperature for 48 h for hematoxylin and eosin (H&E) analysis. The 8 *μ*m tissue sections were stained with hematoxylin and eosin (H&E), using a standard protocol. All tissue sections were examined under a Leica confocal microscope.

### 2.11. Statistical Analysis

Statistical analysis was performed using one-way ANOVA followed by Bonferroni multiple comparison test. *P* < 0.05 was considered statistically significant.

## 3. Results and Discussion

### 3.1. Preparation and Characterization of HANP/HCPT

**Figure 1:**
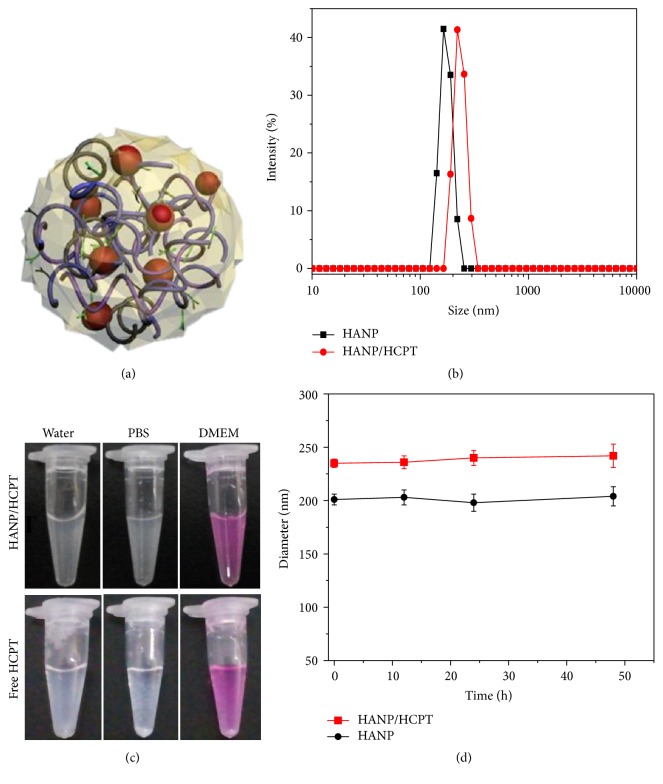
Characterization of HANP/HCPT. (a) Scheme of HANP/HCPT. (b) Dynamic light scattering analysis of HANP and HANP/HCPT. Panels (c) and (d): Stability test of HANP and HANP/HCPT. No precipitation and diameter changes were observed over 48 hours in physiological buffer including water, PBS, and DMEM.

Several classes of topoisomerase inhibitors have been introduced into cancer clinics as potent anticancer drugs. Specifically, a natural indole alkaloid extracted from a Chinese tree* Camptotheca acuminata*, 10-hydroxycamptothecin (HCPT), is a topoisomerase I-specific inhibitor [5, 27]. Previous studies have shown that HCPT and its analogs can stabilize the reversible covalent DNA-Topo-I complex resulting in apoptosis of cancer cells [28]. Unfortunately, clinical application of HCTP is hindered by the poor water solubility and tumor targetability. To improve the tumor targetability and increase the tumor ablation effects of HCPT, we first synthesized hyaluronic acid (HA) nanoparticles (HANP, Figure 1(a)) according to our previous study [8, 9]. The nanoparticles are composed of a hydrophilic outer layer of HA and a hydrophilic inner cavity. Water-insoluble HCPT was physically encapsulated into the hydrophobic cavities by a high pressure homogenizer. This dispersed the HPCT under physiological conditions (Figure 1).

To optimize the encapsulation of HCPT into HANP, different amounts of HCPT (10%, 20%, and 40%) were loaded into HANP, and the loading efficiency was calculated according to the HCPT standard curve (Figure S1 in Supplementary Material available online at https://doi.org/10.1155/2017/4972701).  Table 1 shows that the highest loading efficiency (92.7%) was achieved when 40% of HCPT was applied. We used a HCPT : HANP ratio of 2 : 3 in the following studies.

To verify the encapsulation of HCPT, we compared the size changes before and after loading.  Figure 1(c) shows a 30 nm increase in diameter, which is attributed to the encapsulation of HCPT inside HANP. Compared to free HCPT that immediately precipitated in water, FBS, and cell culture media, the HANP/HCPT showed good dispersion in these buffers. The size of the HANP/HCPT complex did not obviously change with time over two days of incubation at room temperature. These results suggest that HANP/HCPT was successfully constructed with excellent dispersion and stability in physiological buffers suggesting that HCPT was loaded into the interior of the HANP.

### 3.2. In Vitro Cancer Cells Targeting of HANP/HCPT

**Figure 2:**
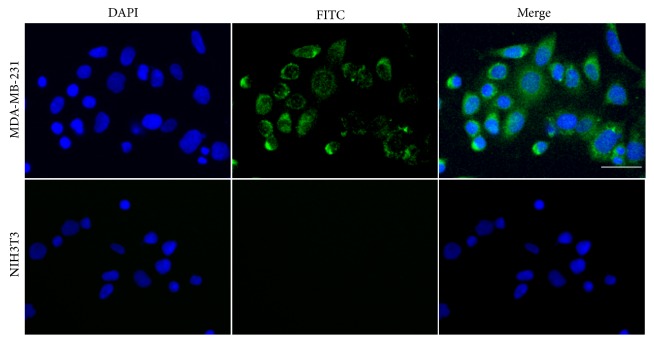
Cell targeting of HANP to MDA-MB-231 and NIH3T3 cells. A strong green color was observed on MDA-MB-231 cells due to the overexpressed CD44, while little HANP was observed on NIH3T3 cells. The HANP was labeled with FITC for the green color. Nuclei were stained in blue. The scale bar is 20 *μ*m.

HA is primary ligand of CD44 and is highly expressed in many diseases including cancer. Biologically, CD44 is a transmembrane glycoprotein that has various functions in cell division, migration, adhesion, and signaling [29]. Recently, the prevalence of CD44 in cancer cell has attracted great attention on its relation to molecular onset of tumor progression [30]. To investigate CD44-mediated cancer cell targeting, we utilized FITC-labeled HANPs (FITC-HANP) for cancer cell labeling.  Figure 2 shows the strong fluorescent signal from FITC-HANP-treated cancer cells. The signals are weak in normal NIH3T3 cells implying that FITC-HANP are labeling the cancer cells. This result indicates that HANP can recognize CD44-overexpressed cancer cells and have potential as a targeted drug carrier for cancer cell growth inhibition.

### 3.3. In Vitro Enzyme-Triggered Drug Release of HANP/HCPT

**Figure 3:**
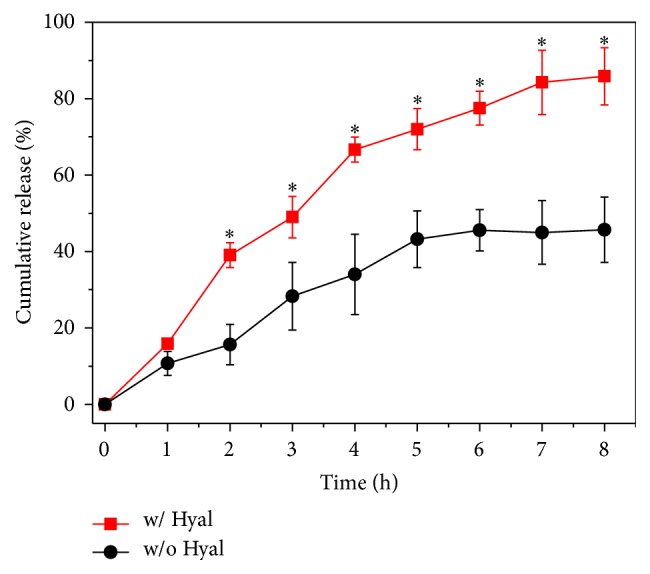
In vitro drug release. In vitro drug release of HANP/HCPT in an acetate buffer (Ph = 4.3, 37°C) with or without hyaluronidase (Hyal). The HCPT quickly released from HANP/HCPT complex in the presence of Hyal, which can degrade HANP. *∗* denotes *P* < 0.05.

We then studied if the payload in the HANP system can be released for cancer treatment. The HCPT release profile was investigated via dialysis experiments.  Figure 3 shows that HCPT was released faster in presence of hyaluronidase (Hyal) than HCPT without Hyal. The cumulative release of HCPT in HANP/HCPT with the presence of native Hyal was 39.05 ± 1.41% in 2 h and only 15.63 ± 5.30% HCPT was detected in buffers without Hyal. This is because the HANP can be destroyed by Hyal via degradation of HA. Thus, the payload is more rapidly released. After 8 h of incubation, 85.84 ± 7.46% of HCPT was released with facility of Hyal; only 45.68 ± 8.54% was released without Hyal. This indicates that the HANP system can release drug in an enzyme-dependent manner. Because native Hyal is overexpressed in tumor tissue, HCPT will mostly be released in tumor and thereby induces only minor side effects on the normal organs.

### 3.4. In Vitro Cytotoxicity of HCPT Loaded HANPs

**Figure 4:**
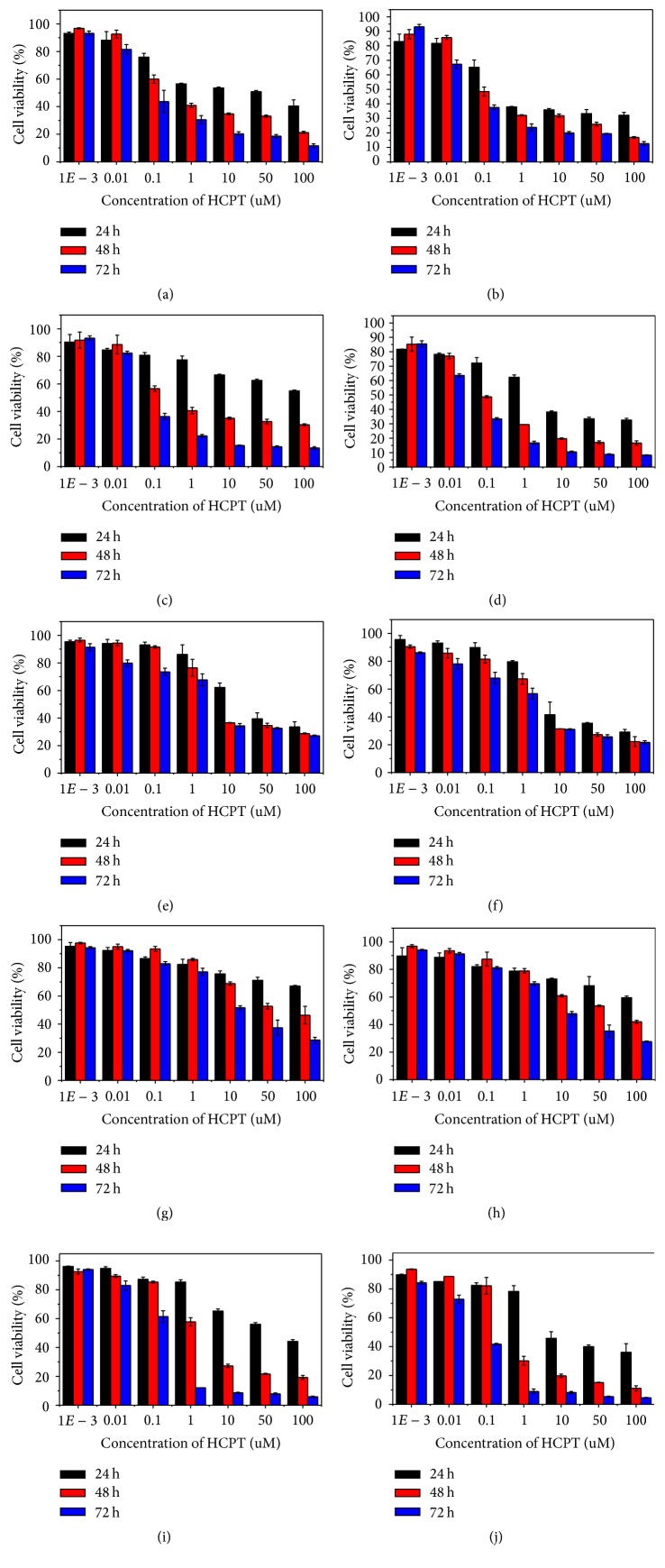
Cytotoxicity of HANP/HCPT. The toxicity of free HCPT and HANP/HCPT was investigated on five different cancer cell lines including MDA-MB-435, MDA-MB-231, HT29, A549, and HepG2. The IC50 was calculated to be 60 nM, 55 nM, 80 nM, 7 *μ*M, and 8 *μ*M, respectively.

In view of excellent cancer cell targeting, we then determined the cytotoxicity of HANP/HCPT in five kinds of cancer cells and one normal cell. The in vitro cytotoxic effects of HANP/HCPT HT29, A549, MDA-MB-231, HepG2, MDA-MB-435, and normal fibroblasts (NIH-3T3) were evaluated using the MTT colorimetric assay (Figure 4). Free HCPT cytotoxicity to these cells was also measured as a control. After 24, 48, and 72 h incubation, both free HCPT and HANP/HCPT showed time-dependent cancer cell toxicity. This effect increased with drug incubation time and induced more cells death. In addition, more cells are killed at higher concentrations of HCPT or HANP/HCPT. Because of the excellent biocompatibility and targetability, the HANP/HCPT demonstrated a 5.38-fold higher IC_50_ than free HCPT in different cell lines (Table 2) indicating that HANP delivered HCPT in an effective way to kill cancer cells in vitro.

### 3.5. In Vivo Antitumor Efficacy of HANP/HCPT

**Figure 5:**
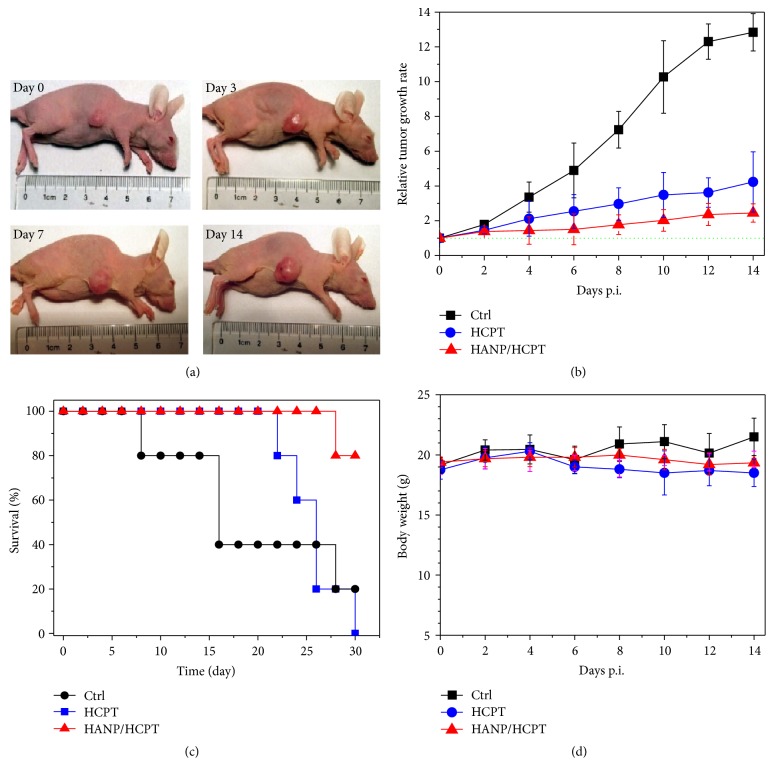
In vivo tumor ablation of HANP/HCPT. (a) Photos of a MDA-MB-231 tumor-bearing mouse model after HANP/HCPT treatment at 0, 3, 7, and 14 days. (b) Growth curve of MDA-MB-231 tumor without treatment and MDA-MB-231 tumor received free HCPT or HANP/HCPT treatments. (c) Survival rate of MDA-MB-231 tumor-bearing mouse model after different treatments. (d) MDA-MB-231 tumor-bearing mouse body weight changes during the study after received different treatment. No obvious changes were found.

Based on the excellent cancer cells targeting and cytotoxicity of HANP/HCPT in vitro, we tested the potency of HANP/HCPT for in vivo cancer treatment. The antitumor efficiency was assessed after intravenous injection of 10 mg/kg HANP/HCPT (equivalent dose of free HCPT). The tumor size changes were measured and recorded. After three rounds of treatment, the nontreated control group and free HCPT treated tumors exhibited a rapid increase in tumor size as a function of time (Figure 5). Notably, no increase in tumor size was found for the HANP/HCPT group (Figures 5(a) and 5(b)). The average tumor size of the treatment group became significantly smaller than that of the control group starting from day 5 (*P* < 0.05) suggesting that tumor growth was inhibited. In terms of survival, 50% of the mice treated with free HCPT died within 20 days due to uncontrollable tumor growth (Figure 5(c)). However, 80% HANP/HCPT treated mice survived for 30 days during the study. The body weight was also recorded as an indicator of the toxicity.  Figure 5(d) shows that the murine body weight values were not changed significantly suggesting negligible toxicity of HANP/HCPT to mice at the doses used here.

### 3.6. 18F FDG PET Predicting and Monitoring the Therapy Response of HANP/HCPT

**Figure 6:**
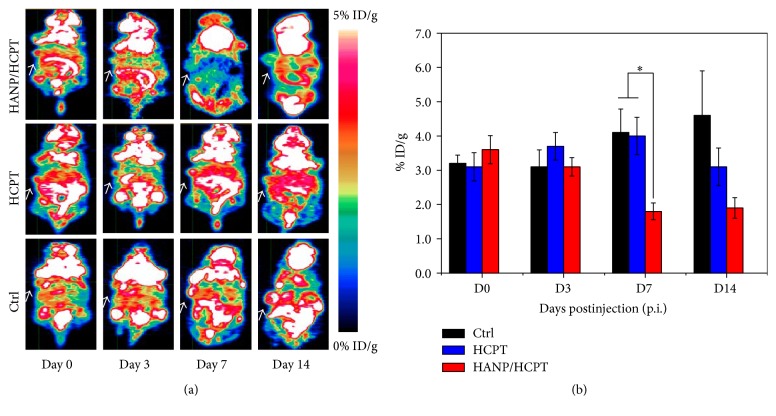
[18F] FDG PET monitoring of HANP/HCPT treatment of MDA-MB-231 tumor. (a) Representative decay-corrected whole-body coronal images of female athymic nude mice with MDA-MB-231 tumors at 1 h after intravenous injection of [18F] FDG on days 0, 3, 7, and 14. (b) Quantitative small-animal PET region-of-interest analysis of tumor uptake of [18F] FDG in each group. *∗* denotes *P* < 0.05.

Tumor cells are very metabolically active and favor the more inefficient anaerobic pathway, which ads to their already increased glucose needs. These combined mechanisms in tumor cells result in a high uptake and retention of FDG versus normal tissue. Because 18F FDG is a glucose analog that behaves as glucose, it will accumulate in tumors and has been widely used for predicting and monitoring therapy responses as an effective PET agent. To monitor the HANP/HCPT tumor treatment response, 18F FDG PET imaging was applied before (day 0) and after treatment (day 3, day 7, and day 14).  Figure 6 shows a prominent increase in [18F] FDG uptake in the control groups treated with saline (3.2 ± 0.24, 3.1 ± 0.49, 4.1 ± 0.69, and 4.6 ± 1.3% ID/g) and free HCPT group (3 ± 0.31, 2.8 ± 0.52, 2.3 ± 0.31, and 1.7 ± 0.4% ID/g) over time to the end of our study. In contrast, [18F] FDG uptake started significantly decreasing from 3.6 ± 0.41% ID/g and 3.1 ± 0.27% ID/g on day 0 and day 3 to 1.8 ± 0.25% ID/g and 1.9 ± 0.29% ID/g on day 7 and day 14 after treatment due to decreased tumor cell metabolic activity suppressed by HANP delivered to HCPT. This cannot be easily differentiated by monitoring tumor size changes.

**Figure 7:**
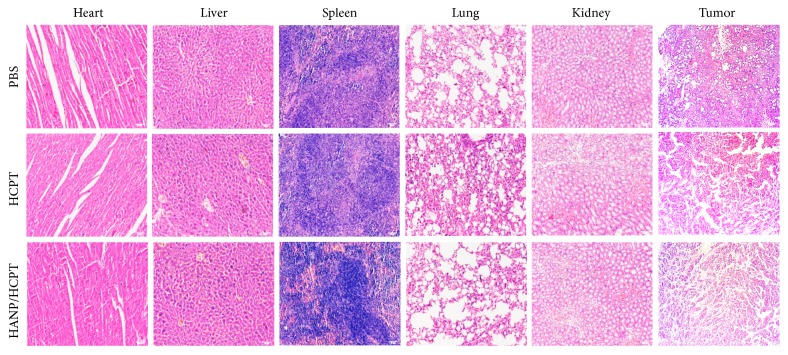
The H&E staining of tumor and major organs. The tumor tissue was significantly destroyed after HANP/HCPT treatment; no abnormalities were seen in the heart, liver, spleen, lungs, or kidneys.

To further confirm the therapeutic effect of HANP/HCPT in tumors, the mice that received different treatments were sacrificed at day 30. The normal organs and tumors were collected and stained with H&E (Figure 7). Histological assessment showed that the tumor tissues were destroyed, and the number of tumor cells were significantly reduced in the HANP/HCPT treated group (Figure 7); tumors in the free HCPT treated group were not affected (similar to the control group) suggesting that HANP delivers HCPT into tumors for tumor growth inhibition. In addition, no histological changes were observed in the normal organs for any treatment groups. This suggests that the HANP/HCPT effectively inhibits tumor growth without affecting nearby normal organs.

## 4. Conclusion

In this report, we successfully constructed a dual targeted nanodrug—hyaluronic acid nanoparticles encapsulated with camptothecin (HANP/HCPT)—for effective therapy of cancer. Compared to free HCPT, the HANP/HCPT showed excellent biocompatibility, tumor cells targeting, and specificity. In the existing of Hyal-2 that is overexpressed in the intracellular compartments of cancer cells, the HANP was disrupted and 85.84 ± 7.46% of HCPT was released in 8 hours. The IC_50_ of HANP/HCPT to MDA-MB-435, HT29, and MDA-MB-231 cells are 60, 80, and 55 nM respectively, which decreased significantly compared to free HCPT. A549 and HepG2 cells were found not sensitive to either HCPT or HANP/HCPT. After intravenous injection of HANP/HCPT into tumor-bearing mice, we measured significant tumor growth inhibition, which is twofold better than free HCPT. The tumor therapeutic potency was also confirmed by [18F] FDG PET, and the early tumor response was detected as early as 7 days after first injection. This will benefit physicians. They can adjust the drug doses and intervals to maintain the antitumor effect and avoid a relapse. The HANP drug carrier can be further extended to other chemotherapy drugs such as SN38. These studies are underway in our group.

## Supplementary Material

Figure S1: Chemical structure of HCPT.
Figure S2: Quantification of HCPT in HANP/HCPT complex.

## Figures and Tables

**Table 1:** HCPT loading efficiency at different conditions.

HCPT : HANP (w/w)	Loading content (wt%)	Loading efficiency (%)
1 : 9	8.56	83.79
1 : 4	18.16	90.82
2 : 3	37.8	92.7

**Table 2:** IC_50_ of HCPT and HANP/HCPT to different cell lines.

Cell types	HCPT (*µ*M)	HANP/HCPT (*µ*M)
MDA-MB-435	0.08	0.06
HT29	0.3	0.08
A549	15	7
MDA-MB-231	0.07	0.055
HepG2	12	8
NIH3T3	>100	>100
