# Supplementary material for: Preclinical Evaluation and Monitoring of the Therapeutic Response of a Dual Targeted Hyaluronic Acid Nanodrug
Source: Contrast Media Mol Imaging. 2017 Jul 11;2017:4972701. doi: 10.1155/2017/4972701 (PMC5612705; doi:10.1155/2017/4972701)
Supplement: Supplementary file 1 — Figure S1: Chemical structure of HCPT. Figure S2: Quantification of HCPT in HANP/HCPT complex. [file 4972701.f1.docx]

**Preclinical Monitoring of the Therapeutic Response of a Dual Targeted Hyaluronic Acid Nanodrug with 18F FDG PET**

Minglong Chen^1, #^, Wenqi Zhang^1,#^, Kai Yuan^2,3^, Mingxiang Bo^4^, Bin Chen^1^, Lu Li^1^, Qingjie Ma^1^, Lei Zhu^1,3,*^, Shi Gao^1,*^

1. Department of Nuclear Medicine, China-Japan Union Hospital, Jilin University, Changchun, China 130033;
2. Department of Breast Surgery, Shandong Provincial Qianfoshan Hospital, Shandong University, Ji’nan, 250012, China;
3. Departments of Surgery, Emory University School of Medicine, Atlanta, Georgia 30322, United States;
4. Pharmaceutical Department, Binzhou Medical University Hospital, Binzhou, China 256603.

# These authors contributed equally to this work.

* To whom correspondence should be addressed. E-mail: [lei.zhu@emory.edu](mailto:lei.zhu@emory.edu) (L.Z.) and [gaoshi800830@163.com](mailto:gaoshi800830@163.com) (S. G.).

**Figure S1.** Chemical structure of HCPT.

**Figure S2.** Quantification of HCPT in HANP/HCPT complex. (a) Absorption peak of HCPT at different concentrations (1, 0.5, 0.25, 0.125, 0.0625 mg/mL) using analytical HPLC at 254 nm. (b) Standard curve of analytical HCPT using HPLC at 254 nm. (c) Absorption peak of 10%, 20% and 40% HANP/HCPT in deionized water.
